# The Sufferings of Christ

**Published:** 1888-03-24

**Authors:** 


### Words of Consolation.

[Communications for this column should be addressed "Chaplain," care of the Editor of The Hospital, who will gratefully welcome any suggestions or inquiries from matrons, nurses, and the staff generally.]

### LXX.—The Sufferings of Christ.

*For Reading to the Sick.*

Knowing that we are redeemed from the outer darkness which remaineth only for those who reject Christ, shall we be altogether ungrateful for such little crosses as may help to identify us with Him? Loneliness of soul, desertion by friends or relations, withdrawal of sympathy, bereavements, seem to whisper at times: "This is hard; but see through this what you have been saved from, if you will." Separation is the bitterest pang we are called to endure here, and we have always the prospect of re-union. Those who are most conscious of God in the world can best endure it; for wherever they are He is always a Presence to them. They are not alone, for the Father is with them. Nevertheless, humanity in its average condition knows of no pain like the pain of being left. How marked this is in children! What can they bear less than being left alone? How marked the same feeling is in ourselves: in our weakest moments, in sickness, just when we are most helpless, to have someone by us, a *presence*—just a hand to hold if we are in pain, or a face to meet our gaze, seeming to understand the sorrow which words have no power to express or explain. Ah! if these comforters ever fail us in the smallest degree, and the sense of desertion is given just a temporary dominion, is it that we are forsaken? Or is there any love to be discerned in the face of the darkness? Surely there is. The light returns, and the heart's quaking ceases, and the miserable fears and horrid temptations which rent the soul asunder are stilled. Almost a taste of very death has been endured. And then once more the Cross stands out so clear against the sky, while He who hangs there seems to say, "I have not forsaken thee. But what of this darkness that thou has felt? what of this passing pain of desolation, this fancied loneliness? Has this given thee any sympathy for Me? Has this set thee meditating, calmly and gratefully, on the rescue which I have secured for thee?" There are some natures too little sensitive, endowed with too little love, ever to feel very keenly here either the absence of sympathy or the loss of friends. They can get on alone; they stand bereavement better than others simply because they do not feel it. There are others so gifted with the sense of God's presence and of the holy angels, so alive to the communion of saints, so full of the invisible world, that they have lost the idea of separation in the victory of their faith. A blessed victory over death indeed! But most of us occupy a position between these two, and we aim at the higher faith of the latter class. Meanwhile, separation in any form but sin may be useful in teaching us the greatness of our salvation. To lose those dear to us, or their esteem; to lose sympathy, or credit, or reputation; to lose all that the world has to give; to be as no one, if need be; nothing of this kind can be counted as loss if it only serve to unite us more closely with the forsaken Jesus in the darkness. There may be much grief in whatever discipline of this kind is ordained for us; there need be no sting. "The sting of death is sin" (1 Cor. xv. 56). The only sting; and why? Because sin is desertion, and death is to be forsaken. "But thanks be to God which giveth us the victory through our Lord Jesus Christ." Learn, therefore, in all periods of loneliness or desolation of spirit to unite yourself with your Saviour on the cross. Then, if some still greater darkness come upon you than you have ever yet known —it may be the darkness of your last hours—you will not think that you are deserted even then. You will be able to feel, "If I say, Surely the darkness shall cover me; even the night shall be light about me" (Psalm cxxxix. 11).

